# *Campylobacter jejuni* biofilms contain extracellular DNA and are sensitive to DNase I treatment

**DOI:** 10.3389/fmicb.2015.00699

**Published:** 2015-07-10

**Authors:** Helen L. Brown, Kate Hanman, Mark Reuter, Roy P. Betts, Arnoud H. M. van Vliet

**Affiliations:** ^1^Gut Health and Food Safety Programme, Institute of Food ResearchNorwich, UK; ^2^Cardiff School of Health Sciences, Cardiff Metropolitan UniversityCardiff, UK; ^3^Campden BRIChipping Campden, UK

**Keywords:** *Campylobacter jejuni*, biofilm, food safety, extracellular matrix, extracellular DNA, antibiotic resistance

## Abstract

Biofilms make an important contribution to survival and transmission of bacterial pathogens in the food chain. The human pathogen *Campylobacter jejuni* is known to form biofilms *in vitro* in food chain-relevant conditions, but the exact roles and composition of the extracellular matrix are still not clear. Extracellular DNA has been found in many bacterial biofilms and can be a major component of the extracellular matrix. Here we show that extracellular DNA is also an important component of the *C. jejuni* biofilm when attached to stainless steel surfaces, in aerobic conditions and on conditioned surfaces. Degradation of extracellular DNA by exogenous addition of DNase I led to rapid biofilm removal, without loss of *C. jejuni* viability. Following treatment of a surface with DNase I, *C. jejuni* was unable to re-establish a biofilm population within 48 h. Similar results were obtained by digesting extracellular DNA with restriction enzymes, suggesting the need for high molecular weight DNA. Addition of *C. jejuni* genomic DNA containing an antibiotic resistance marker resulted in transfer of the antibiotic resistance marker to susceptible cells in the biofilm, presumably by natural transformation. Taken together, this suggest that eDNA is not only an important component of *C. jejuni* biofilms and subsequent food chain survival of *C. jejuni*, but may also contribute to the spread of antimicrobial resistance in *C. jejuni*. The degradation of extracellular DNA with enzymes such as DNase I is a rapid method to remove *C. jejuni* biofilms, and is likely to potentiate the activity of antimicrobial treatments and thus synergistically aid disinfection treatments.

## Introduction

*Campylobacter jejuni* is the most common cause of bacterial foodborne infection within the UK (Nichols et al., 2012). Its success as foodborne pathogen contrasts with its fastidious nature, as it requires specific atmospheric conditions, nutrient-rich growth medium and has a narrow temperature range (between 35 and 45°C) for growth. Several mechanisms for survival in the food chain have been proposed, including the ability of *C. jejuni* to enter a viable but none culturable (VBNC) state (Rollins and Colwell, 1986), as well as formation of *de novo* biofilms or integration into existing (multispecies) biofilms (Teh et al., 2014). Biofilms are defined as surface attached populations, either single or multiple species, which are surrounded by a self-produced extracellular matrix (Donlan, 2002). The extracellular matrix differs depending on the species within the biofilm but typically comprises of DNA, proteins and polysaccharides (Branda et al., 2005).

The extracellular matrix is an essential component of bacterial biofilms, and usually accounts for more than 90% of the dry mass of a biofilm (Flemming and Wingender, 2010). It allows cells to remain hydrated and metabolically active by trapping nutrients and liquid near the bacterial cells. It also reduces access of larger molecules such as antimicrobials (Mulcahy et al., 2008; Billings et al., 2013), leading to increased bacterial persistence, and is structurally important, maintaining the shape of the biofilm and ensuring the cohesion of the biofilm (Sutherland, 2001). Extracellular DNA (eDNA) appears to have a structural role in the biofilms of many different species, including *Pseudomonas aeruginosa* (Chiang et al., 2013), *Staphylococcus aureus* (Mann et al., 2009), *Listeria monocytogenes* (Harmsen et al., 2010), and *Escherichia coli* (Zhao et al., 2013).

Recent studies have shown that eDNA is important for biofilm establishment and maintenance by *C. jejuni* strain 81–176 in laboratory conditions (Bae et al., 2014; Svensson et al., 2014), but this has not yet been studied in the context of the conditions encountered by *C. jejuni* in the processing environment. Previous studies have shown that food chain relevant conditions such as atmospheric oxygen levels (Reuter et al., 2010), reduced temperatures (Buswell et al., 1998) and surface soiling (Brown et al., 2014) all increase *C. jejuni* biofilm formation, and as such may also influence the composition of the *C. jejuni* biofilm, necessitating the study of *C. jejuni* biofilms in these conditions.

The aim of this study was to further investigate the role of eDNA within the *C. jejuni* biofilm, with particular reference to its role in food chain relevant environments. Here we present evidence that eDNA is also present in biofilms of *C. jejuni* reference strains NCTC 11168 and 81116 when incubated in aerobic conditions and on food chain relevant surfaces such as stainless steel. Degradation of eDNA by DNase I leads to a rapid loss of biofilm structure, releasing cells into the planktonic phase. Treatment of surfaces with DNase I also inhibits *de novo* biofilm formation, either due to re-growth from single, attached, cells or from *de novo* attachment of *C. jejuni* cells. Addition of *C. jejuni* DNA to biofilms results in the transfer of genetic markers, which can contribute to spread of antimicrobial resistance in *C. jejuni* populations.

## Materials and methods

### *C. jejuni* strains and growth conditions

*C. jejuni* strains NCTC 11168 (Parkhill et al., 2000), its derivative expressing a green fluorescent protein and chloramphenicol resistance marker (*C. jejuni* NCTC 11168 *cj0046::gfp*-Cm^R^) (Brown et al., 2015), strain 81116 (Pearson et al., 2007) and all microaerobic biofilm incubations were routinely cultured in a MACS-MG-1000 controlled atmosphere cabinet (Don Whitley Scientific) under microaerobic conditions (85% N_2_, 5% O_2_, 10% CO_2_) at 37°C. For growth on plates, strains were either grown on Brucella agar or BAB with Skirrow antibiotic supplement (10 μg/ml vancomycin, 5 μg/ml trimethoprim, 2.5 IU polymyxin-B). Broth culture was carried out in Brucella broth (Becton & Dickinson).

### *Campylobacter* growth for biofilm assay

Frozen 50 μl single-use glycerol stocks were thawed, inoculated onto Skirrow plates and grown overnight at 37°C in microaerobic conditions (5% O_2_, 10% CO_2_ and 85% N_2_). Cells from the Skirrow plate were used to inoculate Brucella broth then grown overnight as a shaking culture (37°C, microaerobic conditions). Following overnight growth, cell cultures were adjusted to an A_600_ of 0.05 in Brucella medium or Brucella medium supplemented with 5% v/v chicken juice. To allow biofilm formation, 1 ml of this solution was added to either a sterile borosilicate glass test tube (Corning) or 3 ml to a six-well polystyrene tissue culture plate (Corning) containing a sterile stainless steel coupon (Stainless steel type 1.4301 according to EN 10088-1, with a Type 2B finish according to EN 10088-2). In each biofilm assay a test tube containing sterile Brucella medium was incubated alongside the *C. jejuni* containing tubes to ensure sterility was maintained and, following crystal violet staining, to quantification of staining levels where biofilm was not present. Tubes were incubated at 37°C in atmospheric air conditions using an Innova 4230 (New Brunswick Scientific) incubator at 37°C. Unless otherwise stated all biofilms were formed in aerobic conditions at 37°C for 48 h before staining procedures were carried out. For each assay a microaerobic biofilm control was also undertaken, to ensure that oxygen availability does not have a major effect on results and to allow comparison with previous studies (Reuter et al., 2010; Brown et al., 2013, 2014, 2015). This sample was prepared in exactly the same way as the aerobic biofilm cultures but test tubes were placed back in the 37°C microaerobic incubator for all static incubations.

### Preparation of chicken juice

Chicken juice was prepared as described previously (Brown et al., 2013, 2014). Briefly, frozen commercially available whole chickens were purchased from UK supermarkets before thawing at room temperature. Exudate was collected, centrifuged to remove debris and sterilized by using a 0.2 μm sterile polyethersulfone (PES) syringe filter (Millipore) before aliquotting and storage at −20°C until use. Chicken juice was diluted v/v in Brucella medium for use in biofilm assays.

### Enzyme treatment of *C. jejuni* biofilms

For DNase I treatments, unless otherwise stated, a volume of 4 μl DNase I enzyme (Fermentas), giving a final concentration within the biofilm of 4 U/ml v/v and 4 μl of DNase I buffer (Fermentas) were added to each test tube, along with 1 ml of diluted cell suspension at either the start of the static incubation or after 12, 24, 36, or 48 h of static incubation. Following treatment, static cultures were placed back in 37°C, aerobic conditions to complete the 48 h incubation before staining with crystal violet to allow biofilm quantification. For restriction enzyme digest of biofilms 4 μl of 10 U/μl *Bam*HI, *Blp*I, *Hae*III, *Hin*dIII, *Msc*I or *Rsa*I, (NEB), or DNase I (Fermentas), or RNase (QIAGEN) were added to test tubes containing diluted *C. jejuni* suspension prior to static incubation and then incubated at 37°C for 48 h in aerobic conditions. Equal volumes (4 μl) of the buffers and bovine serum albumin were also added if recommended by the manufacturers. For the assays assessing the time required for DNase I activity, biofilms were allowed to form for 48 h before addition of 4 U/ml v/v DNase I enzyme (1 U/μl, Fermentas) and 4 μl of DNase I buffer to the samples, followed by incubation for up to 2 h. During the incubation with enzyme the samples were placed in 37°C, aerobic conditions. All samples were subsequently stained with crystal violet.

For assessment of biofilm regrowth, biofilms were allowed to establish for 48 h followed by a 15 min incubation with DNase I. Tubes were then washed twice with sterile PBS followed by addition of either an equal volume of bacterial culture with an A_600_ of 0.05, or sterile Brucella medium, followed by a further 48 h incubation at 37°C in aerobic conditions. All samples were subsequently stained with crystal violet. In order to ensure consistency between control and treatment samples all tubes were manipulated in exactly the same way, being removed and placed back in the same incubation conditions during each enzyme addition. Heat inactivated DNase I was prepared by incubating an aliquot of DNase I and its buffer at 95°C for 10 min and allowing to cool before addition to the biofilm cultures.

### Visualization of extracellular DNA from shaking cultures and biofilms

Following incubation to allow biofilm formation in both aerobic and microaerobic conditions, the supernatant was removed and the tubes were rinsed once with sterile PBS to remove loosely attached bacterial populations. After rinsing and removal of the rinse suspension a second 1 ml volume of sterile PBS was added to each test tube and a sterile cotton wool swab was used to gently swab to walls of the test tube, releasing the biofilm from the walls of the test tube and in to suspension. The resuspended biofilm (PBS containing the loosened biofilm cells) and supernatant (liquid initially contained within the test tube) from several biofilm cultures were collected and pooled before diluting to a A_600_ of 0.3. Aliquots were mixed with gel loading buffer (NEB) and added to a 0.9% agarose gel and run at 100 V for 45 min in 0.5% TBE buffer. A 1 kb ladder (NEB) was used for size comparison. Following electrophoresis, nucleic acids were stained using ethidium bromide, and DNA was visualized using a GelVue UV light and documented using a U:Genius gel documentation system (Syngene). The amount of eDNA in planktonic and biofilm fractions was quantified by comparing the intensity of the DNA bands after UV illumination and comparison with the 3 kb marker fragment (125 ng), using ImageJ (Rasband, W.S., ImageJ, U. S. National Institutes of Health, Bethesda, Maryland, USA, http://imagej.nih.gov/ij/, 1997–2014). Quantification was based on three independent experiments.

### Restriction digest of *C. jejuni* genomic DNA

A 1 μl volume of restriction enzyme (*Bam*HI, *Blp*I, *Hae*III, *Hin*dIII, *Msc*I, or *Rsa*I, all supplied by NEB), or DNase I (1U/ μl, Fermentas), or RNase (QIAGEN) was added to a mixture containing ~500 ng of *C. jejuni* NCTC 11168 or 81116 genomic DNA, prepared using a commercial kit (QIAGEN) following manufactures guidelines, 1 μl of 10× enzyme buffer (if required), 1 μl of 1 mg/ml BSA (if required) and molecular grade water to a final volume of 10 μl. Samples were incubated for 60 min in a 37°C water bath to allow digestion of the genomic DNA. DNA was visualized using a GelVue UV light and documented using a U:Genius gel documentation system (Syngene).

### Assessment of natural transformation within the biofilm

Genomic DNA was extracted from the *C. jejuni* NCTC 11168 *cj0046::gfp*−Cm^R^ mutant (Brown et al., 2015) using a commercial kit (QIAGEN), following manufacturers guidelines. DNA concentration was calculated after the final elution and stored at −20°C until use. The standard 48 h static biofilm incubation was carried out, using duplicate test tubes for all conditions. A total of 2 μg genomic DNA was added to test tubes either prior to the start of biofilm incubation, or following 24 h of static incubation. Following a total of 48 h of incubation one test tube of each condition was stained using crystal violet and the second tube washed once with 1 ml PBS and the biofilm population released by swabbing with a sterile cotton bud. Both the supernatant and released biofilm population were retained for viability assessment.

### Crystal violet staining

Cell suspensions were removed from the test tubes before washing with distilled water before drying at 60°C for 30 min. A 1 ml of 1% w/v crystal violet solution was added and tubes were further incubated on a rocker at room temperature for 30 min. After this incubation, the non-bound dye was removed from the tubes by thorough washing in water followed by drying at 37°C. Bound crystal violet was dissolved by adding 20% acetone/80% ethanol and incubating on a rocking platform for 15 min at room temperature. The resulting dissolved dye was measured at a wavelength of 590 nm using a Biomate 5 spectrophotometer (Thermo Scientific).

### 2,3,5 triphenyltetrazolium chloride (TTC) staining

This method was carried out as previously described (Brown et al., 2013, 2014). Following a 48 h incubation to allow biofilm formation, cell suspensions were removed and test tubes were washed twice with 1 ml of sterile PBS. A 1.2 ml volume of Brucella broth supplemented with 0.05% w/v TTC was then added to each test tube before further incubation at 37°C in microaerobic conditions for 72 h. Following secondary incubation, the TTC solution was removed and the test tubes were air dried. Bound TTC dye was dissolved as above using 20% acetone/80% ethanol and the A_500_ of the solution measured.

### Assessment of cell viability by culture

To determine the number of viable cells, the planktonic fraction, or released biofilm population was 10-fold serially diluted eight times in PBS and 5 μl of each dilution spotted on Brucella agar plates or (for assessment of natural transformation) Brucella agar containing 10 μl/ml chloramphenicol. After 2 days of growth at 37°C in microaerobic conditions, the dilution resulting in two or more colonies was recorded. Cell viability in biofilm assays was assessed upon initial addition of cultures into static culture and following static incubation, prior to crystal violet staining and, where necessary, following the 72 h TTC incubation.

### Statistics

Statistical analysis was carried out using GraphPad Prism software. At least three biological replicates (each with three technical replicates unless otherwise stated) were used to calculate mean and standard deviation. Significance was measured using either a Mann–Whitney test (biofilm formation) or ANOVA (DNA yield).

## Results

### Extracellular DNA is present within the *C. jejuni* biofilm and during both aerobic and microaerobic incubation

**Figure 1:**
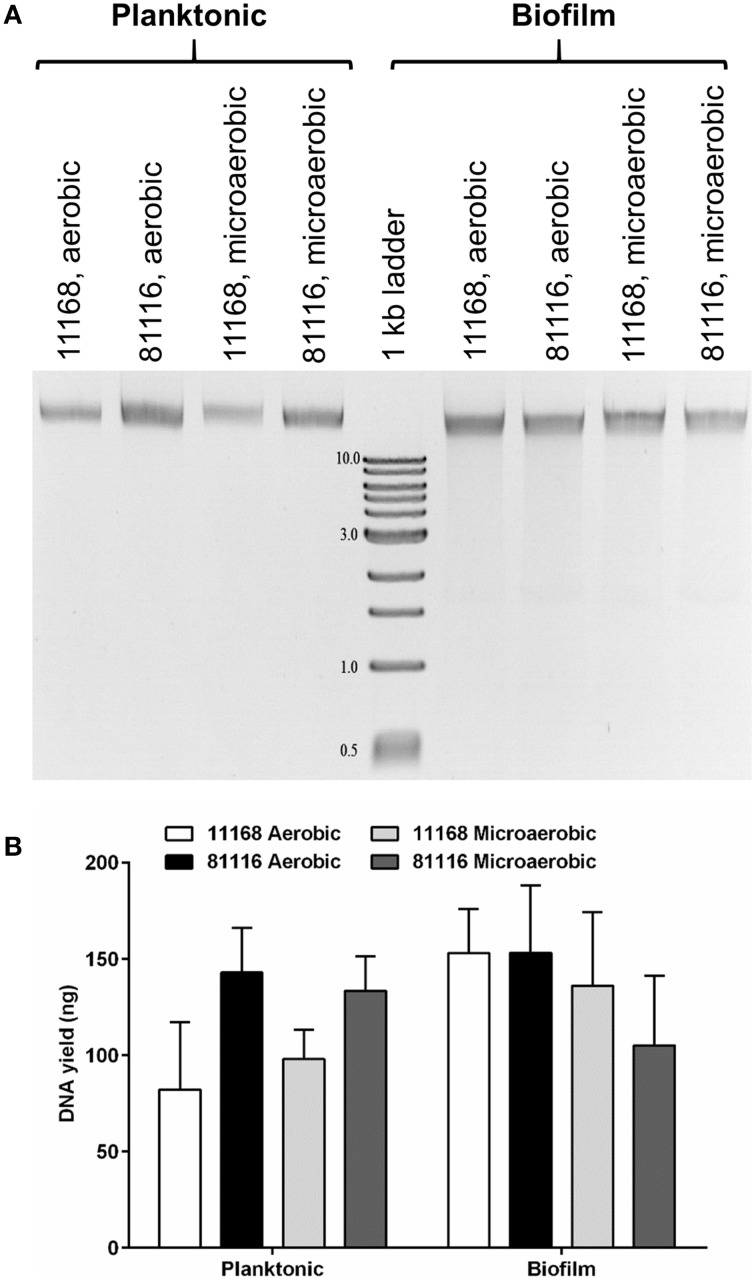
**Extracellular DNA is present in *C. jejuni* biofilms incubated in either aerobic and microaerobic conditions**. **(A)** A representative agarose gel showing both planktonic and adhered cells recovered from 48 h static cultures and loaded directly on a 0.9% agarose gel. Following gel electrophoresis, DNA was detected using ethidium bromide and UV light. Numbers next to 1 kb marker bands indicate the size of the fragments in kilobases. **(B)** DNA was quantified (*n* = 3) by comparing the intensity of the DNA bands with the 3 kb fragment (125 ng) using ImageJ. Error bars show standard deviation. There is no statistically significant difference in DNA yield between the strains or culture conditions as determined using ANOVA.

Biofilms of *C. jejuni* NCTC 11168 and 81116 were generated and used for the investigation of eDNA. Separation of nucleic acids on agarose gels showed the presence of extracellular DNA in both the biofilm and planktonic fractions, independent of whether the biofilm samples were incubated in aerobic or microaerobic conditions (Figure 1). Within the biofilm samples, there was no distinguishable difference between the eDNA bands produced by *C. jejuni* NCTC 11168 and 81116, although in the supernatant, *C. jejuni* NCTC 11168 cultures contained less DNA than *C. jejuni* 81116. The atmospheric conditions used for the incubation did not seem to affect eDNA levels, although as previously reported, total biofilm mass increased during aerobic biofilm incubation (Reuter et al., 2010; Brown et al., 2014).

### Addition of DNase I leads to rapid reduction of biofilm levels and prevents formation of new biofilms

**Figure 2:**
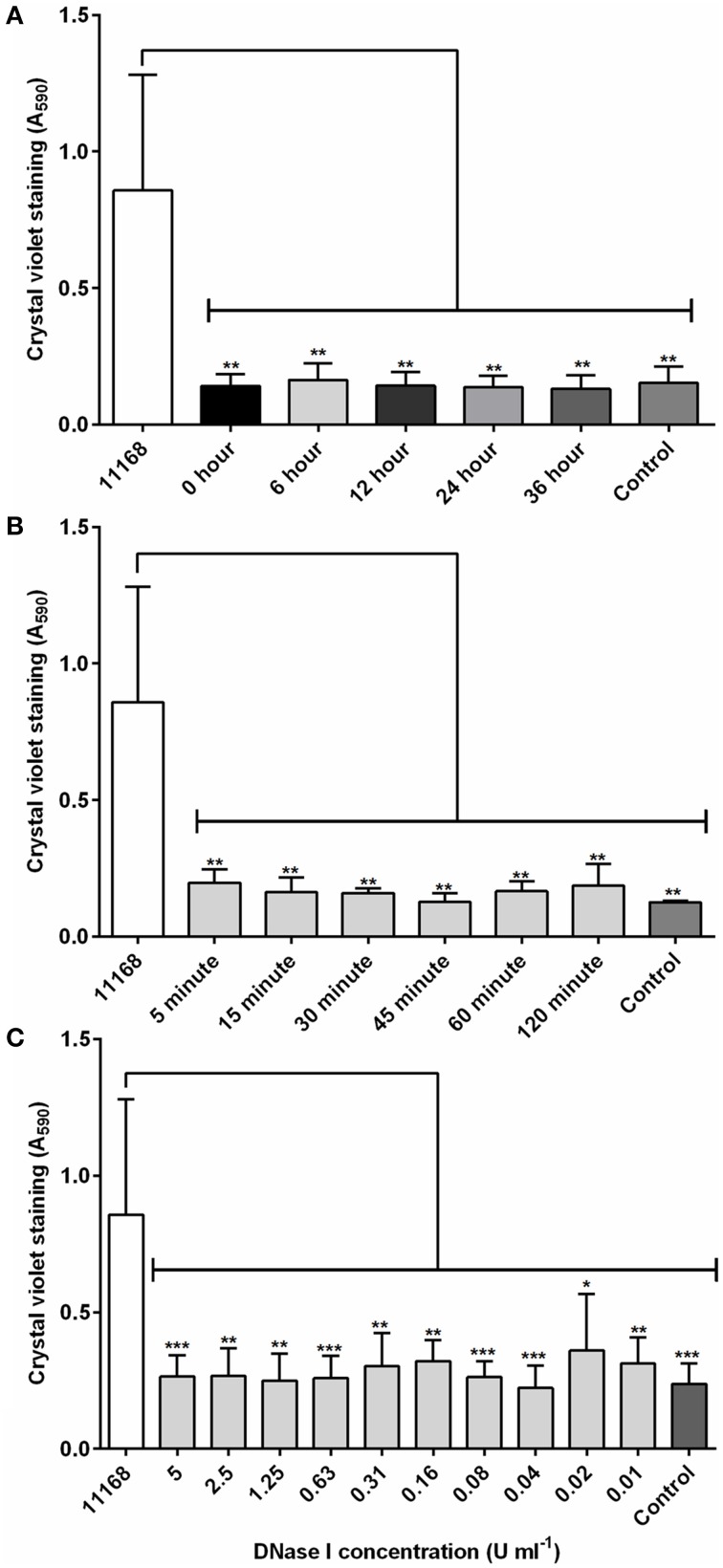
**DNase I is able to rapidly degrade *C. jejuni* NCTC 11168 biofilms**. **(A)** DNase I (4 units/ml) was added at defined intervals to aerobically incubated NCTC 11168 cultures over a 48 h static incubation and biofilm degradation assessed by crystal violet staining. **(B)** Following a 48 h static incubation to allow biofilm formation, DNase I was added to biofilms for between 5 and 120 min before biofilm degradation was assessed. **(C)** The concentration of DNase I required for biofilm control was also assessed using DNase I concentrations of between 0.01 and 5 U/ml. In each graph, “11168” represents an untreated biofilm culture of *C. jejuni* NCTC 11168 and “control” represents a tube containing sterile Brucella medium only. Error bars show standard deviation. Statistically significant results, as determined using the Mann–Whitney U test, are indicated using an asterisk (^*^ *P* < 0.05, ^**^ *P* < 0.01, ^***^ *P* < 0.001).

To assess whether the role of eDNA differs between different stages of biofilm maturity in *C. jejuni*, DNase I was added at 12 h intervals over the total of a 48 h incubation in aerobic conditions. There was no detectable *C. jejuni* biofilm after incubation with 4 U/ml DNase I, regardless of the age of the biofilm (Figure 2A), indicating that eDNA is an important extracellular matrix component during both initial attachment and maturation. We next assessed how rapidly degradation occurs by treating biofilms grown for 48 h with DNase I followed by detection with crystal violet at timed intervals over a two h period. Following only a 5 min incubation with DNase I, there was no detectable staining on the glass surface, and A_590_ values were indistinguishable from the negative control containing Brucella medium only (Figure 2B). Levels of staining did not reduce further at later time points, suggesting that a 5 min treatment can achieve degradation of the eDNA in the *C. jejuni* biofilm and results in a reduction of biofilm levels below the detection limit (Tresse et al., 2006).

Finally, the concentration of DNase I required to degrade the biofilm was also investigated. Addition of DNase I at concentrations ranging from 0.01 to 5 U/ml were significantly reduced crystal violet staining, and there was no statistically significant difference between DNase I treated test tubes and the negative control tube containing Brucella medium only (Figure 2C). It is interesting to note that DNase I treatment had no impact on cell viability, and most likely only degrades the biofilm matrix, resulting in the release of attached cells into suspension. Biofilms incubated with DNase I in microaerobic conditions also showed the same pattern, confirming that the effects observed were not a response to atmospheric condition, but DNase I treatment. Inactivation of DNase I by heat treatment removed its ability to affect C. *jejuni* biofilms (Figure S1), but did not inhibit growth of *C. jejuni*.

**Figure 3:**
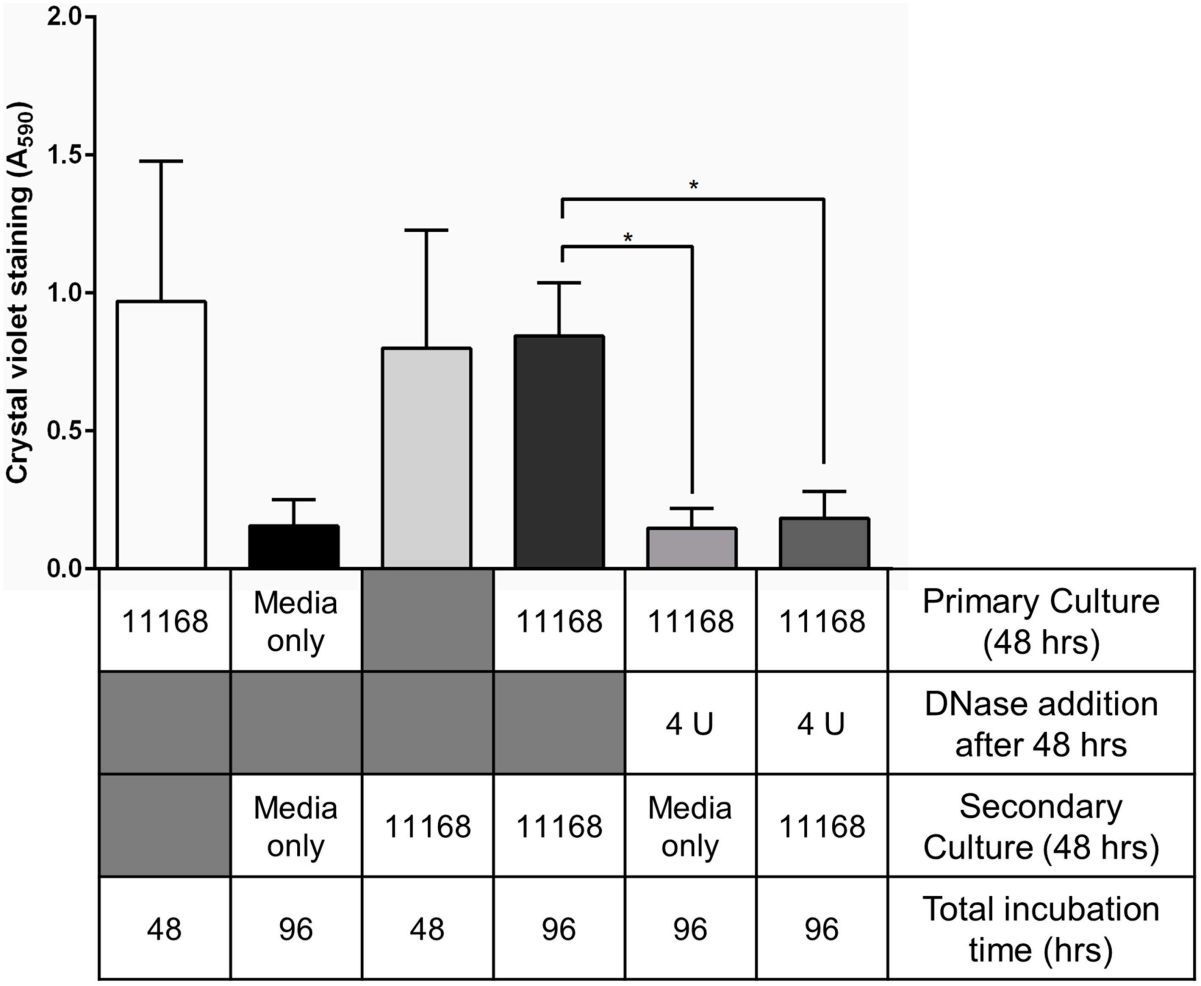
**Treatment of pre-existing biofilms with DNase I leads to inhibition of biofilm regrowth**. *C. jejuni* NCTC 11168 biofilms were allowed to form for 48 h in sterile borosilicate glass test tubes. To assess biofilm re-growth following DNase I treatment, two sets of tubes were treated with 4 U/ml DNase I for 15 min then washed with sterile PBS. Tubes were then supplemented with either fresh Brucella media (fifth bar) or fresh *C. jejuni* NCTC 11168 culture (sixth bar) and incubated for a further 48 h. The following controls were also prepared: *C. jejuni* NCTC 11168 biofilm formation following primary culture (first bar, white), tubes supplemented with sterile Brucella media (second bar, black), *C. jejuni* NCTC 11168 biofilm formation following only secondary culture (third bar, light gray), and 48 h-old *C. jejuni* NCTC 11168 biofilm, washed with PBS, then supplemented with fresh *C. jejuni* NCTC 11168 culture (fourth bar, dark gray). Error bars show standard deviation. Statistically significant results, as determined using the Mann–Whitney U test, are indicated using an asterisk (^*^ *P* < 0.05).

The long-term effects of DNase I-mediated degradation of *C. jejuni* biofilms from abiotic surfaces was assessed by adding fresh *C. jejuni* NCTC 11168 culture to DNase I-treated and washed borosilicate test tubes previously containing a *C. jejuni* biofilm. There was no detectable *C. jejuni* biofilm in either the tubes with added Brucella medium or the tubes with added *C. jejuni* in either aerobic or microaerobic conditions (Figure 3). This suggests that DNase I treatment is not only a rapid method of degrading *C. jejuni* NCTC 1168 biofilms but also prevents biofilm regrowth.

### Restriction digestion of eDNA leads to reduced levels of *C. jejuni* biofilm

**Figure 4:**
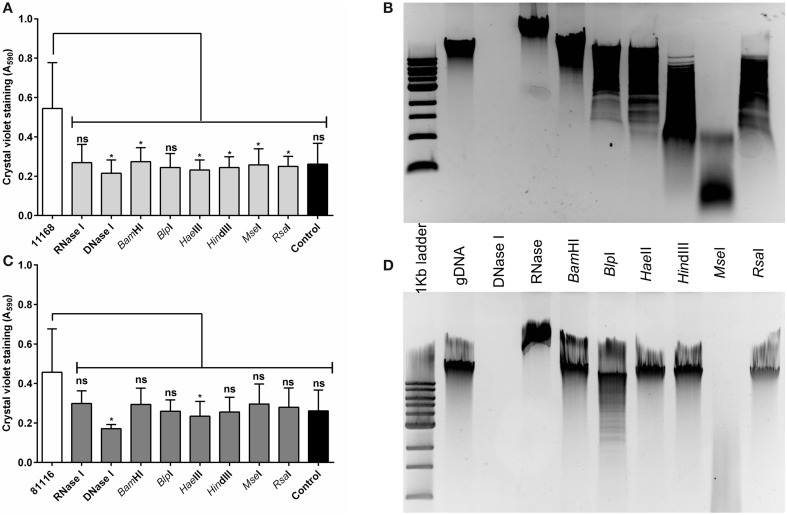
**Restriction endonuclease treatment of *C. jejuni* biofilms reduces biofilm formation**. Static cultures of *C. jejuni* NCTC 11168 **(A,B)** and 81116 **(C,D)** were prepared then supplemented with either DNase I, RNase, or a single restriction endonuclease. Cultures were incubated for 48 h at 37°C in aerobic conditions. A range of restriction enzymes was selected, based on varying levels of DNA fragmentation following digestion of *C. jejuni* NCTC 11168 **(B)** and 81116 **(D)** genomic DNA. Restriction enzyme and DNase I treatment of NCTC 11168 biofilms lead to a reduction in biofilm formation. The same trend was observed for *C. jejuni* 81116, although only DNase I and *Hae*III digestion were significantly different from the control. Error bars show standard deviation. Statistically significant results, as determined using the Mann–Whitney U test, are indicated using an asterisk (^*^ *P* < 0.05).

The eDNA found within the *C. jejuni* NCTC 11168 and 81116 biofilms is of high molecular weight (Figure 1), and we speculated that biofilm formation requires high molecular weight nucleic acids, rather than simply the presence of any nucleic acids. Six restriction enzymes were selected, which are predicted to digest *C. jejuni* genomic DNA to a range of fragment sizes (Figures 4C,D), and these enzymes were assessed for their ability to degrade 48 h old *C. jejuni* biofilms. With *C. jejuni* NCTC 11168 there was a significant reduction in crystal violet staining for all six restriction enzymes tested, with little variation between enzyme treatment and the negative control (Figure 4A). Although the same trend was observed with *C. jejuni* 81116 biofilms, this was not statistically significant except for DNase I treatment (Figure 4B). This was consistent with the reduced digestion observed with *C. jejuni* 81116 genomic DNA, producing fragments of higher molecular weight than those obtained by digestion of *C. jejuni* NCTC 11168 genomic DNA (Figure 4D).

### DNase I treatment is also effective on food chain relevant surfaces

**Figure 5:**
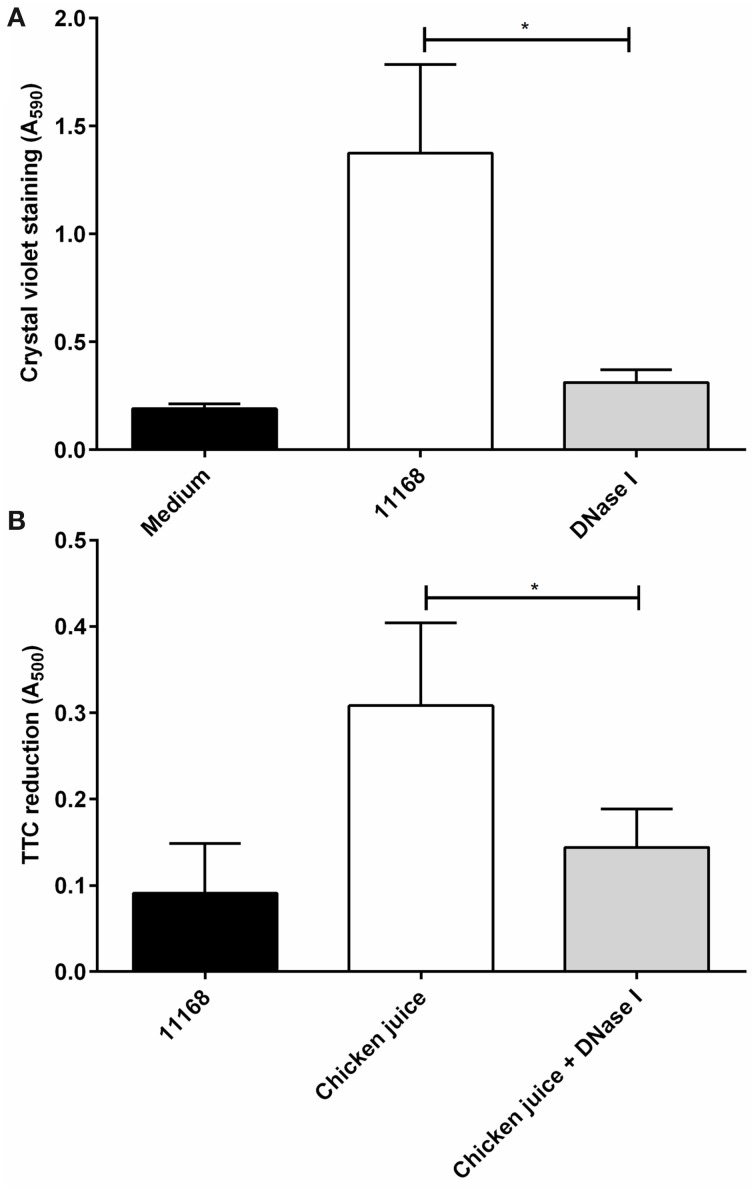
**DNase I treatment is effective against *C. jejuni* biofilms on stainless steel surfaces and in the presence of organic materials in aerobic conditions**. The ability of DNase I to inhibit biofilm formation of *C. jejuni* NCTC 11168 on sterile, stainless steel coupons **(A)** or in the presence of chicken juice, mimicking a conditioned surface **(B)**. TTC staining was used to measure biofilm formation in the presence of chicken juice **(B)**. DNase I is able to significantly decrease biofilm formation in both conditions. Error bars show standard deviation. Statistically significant results, as determined using the Mann–Whitney U test, are indicated using an asterisk (^*^ *P* < 0.05).

The effect of DNase I treatment on *C. jejuni* biofilms formed on food-relevant surfaces such as stainless steel (Somers et al., 1994; Thormar and Hilmarsson, 2010), and on heavily soiled surfaces (De Cesare et al., 2003; Brown et al., 2014) was assessed using *C. jejuni* NCTC 11168 biofilms formed on sterile stainless steel coupons. There was a significant reduction of crystal violet staining following DNase I treatment (Figure 5A). Crystal violet staining of the coupons showed no detectable biofilm following static aerobic incubation in the presence of DNase I, however significant levels of biofilm formation were observed when DNase I was not present (Figure 5A). In order to mimic environments where heavy soiling occurs, *C. jejuni* NCTC 11168 cultures were incubated statically in Brucella medium containing 5% v/v chicken juice. Chicken juice is a complex, undefined exudate obtained from defrosted whole chickens (Birk et al., 2004, 2006) and has a high protein and lipid content, and its presence results in increased biofilm formation due to its ability to condition abiotic surfaces (Brown et al., 2014). DNase I treatment of biofilms formed in the presence of 5% v/v chicken juice did result in a significant reduction of staining compared to untreated biofilms, although there was some residual staining, suggesting that on heavily soiled surfaces DNase I treatment does not provide the same level of biofilm degradation as observed in culture medium only (Figure 5B).

### Biofilms allow genetic transfer of antibiotic resistance to *C. jejuni*

**Figure 6:**
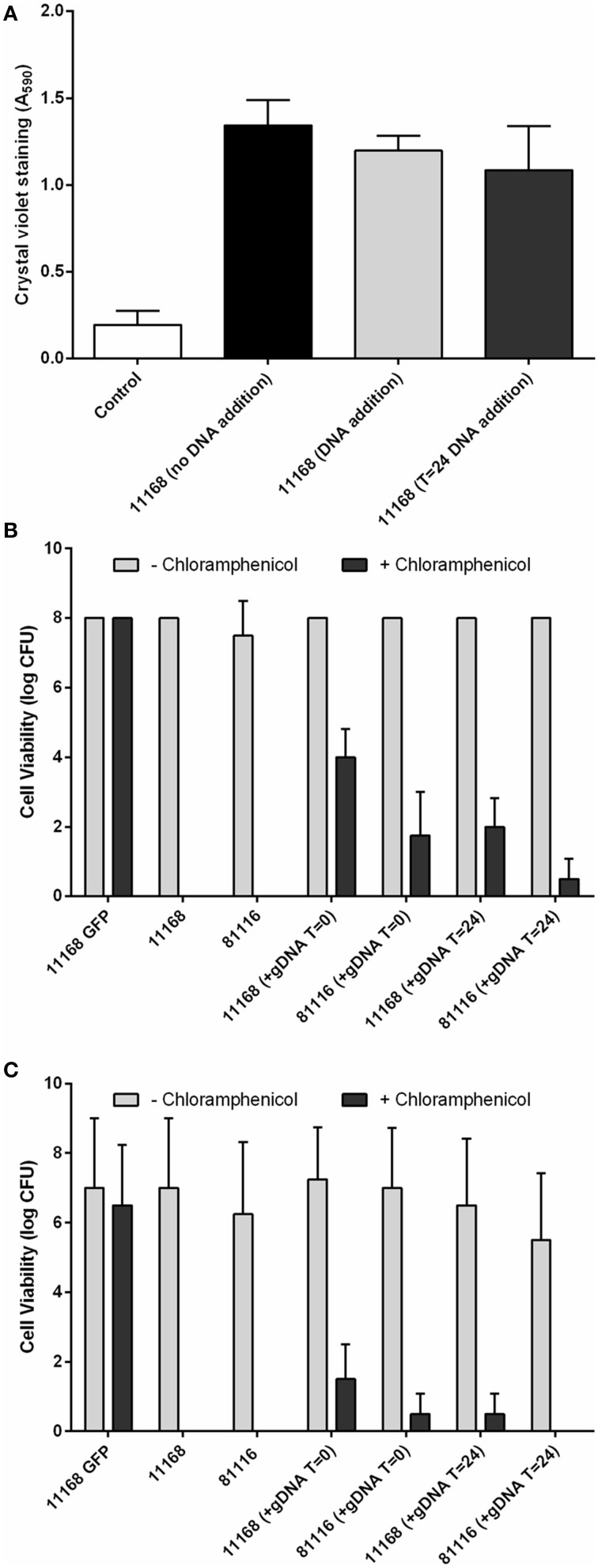
***C. jejuni* NCTC 11168 and 81116 can acquire genetic traits from exogenous DNA during static incubation**. *C. jejuni* NCTC 11168 and 81116 biofilms were allowed to develop for 48 h in the presence of 2 μg *C. jejuni* NCTC 11168 *cj0046*::*gfp*+-CmR genomic DNA. Supplementation with eDNA did not lead to changes in biofilm formation **(A)**. Plating both planktonic **(B)** and biofilm **(C)** cells on both Brucella media and Brucella media supplemented with 10 μg/ml chloramphenicol shows emerging chloramphenicol resistant cells suggesting integration of the chloramphenicol resistance gene, via natural transformation, into the genomes of both planktonic and biofilms cells. Error bars show standard deviation.

Given the presence and structural importance of the eDNA we hypothesized that addition of exogenous DNA may further increase biofilm formation. This was tested by the addition of 2 μg of genomic DNA, isolated from a *C. jejuni* NCTC 11168 strain expressing a GFP protein and containing an antibiotic resistance marker. Addition of genomic DNA did not lead to significant differences in the levels of crystal violet staining (Figure 6A). This indicates that although eDNA is essential for biofilm formation and structural stability, in contrast to previous research on *C. jejuni* 81–176 biofilms (Svensson et al., 2009, 2014), exogenous DNA does not act synergistically with eDNA within the *C. jejuni* NCTC 11168 and 81116 biofilms.

While exogenous genomic DNA was not able to increase biofilm formation, genetic transfer of the antibiotic resistance marker was detected in both the planktonic and biofilm-associated cells (Figures 6B,C). Chloramphenicol-resistant colonies were recovered from both planktonic and biofilm phases following addition of *C. jejuni* NCTC 11168 *cj0046::gfp*−Cm^R^ genomic DNA to static cultures of the wild-type NCTC 11168 and 81116 strains. No resistance was observed in cultures not containing *C. jejuni* NCTC 11168 *cj0046::gfp*−Cm^R^ genomic DNA, suggesting that neither planktonic or biofilm cultures of strains NCTC 11168 or 81116 are naturally resistant to chloramphenicol at the levels used in these assays (10 μg/ml). Where genomic DNA had been added to the suspension at the start of static incubation, resistant cells were present in both planktonic (Figure 6B) and biofilm (Figure 6C) cultures. In cultures where genomic DNA had been added at a later (24 h) time point, lower levels of chloramphenicol-resistance were observed (Figures 6B,C).

## Discussion

Microbial biofilms constitute an important problem for the food industry. There is an increasing body of evidence that biofilms can aid survival of *C. jejuni* in the food chain. *C. jejuni* has previously been shown to form both single (Joshua et al., 2006) and multispecies (Sanders et al., 2007) biofilms, and biofilm formation has also been demonstrated on food chain relevant materials such as stainless steel (Peyrat et al., 2008; Sanders et al., 2008; Brown et al., 2014) and in food chain relevant environmental conditions (Reuter et al., 2010; Brown et al., 2014). While the phenomenon of biofilm formation is well established for *C. jejuni*, there is less information available on the composition and role of the extracellular matrix in the processing environment. Biofouling of surfaces is a problem within the food industry, where organic materials are present, and areas of attention have not only been on antimicrobial treatment, but also on biofilm dispersal and prevention. Combination treatment including various enzymatic treatments, surfactants and chelating agents may provide a suitable alternative to the chemical treatments currently in use for biofilm degradation within food processing areas (Lequette et al., 2010). The use of DNase I is an example of one such enzymatic treatment.

Treatment of biofilm-based bacterial infections with DNases has increased in recent years, and the human recombinant DNase dornase alpha (Pulmozyme) is now frequently used in the treatment of cystic fibrosis (Konstan and Ratjen, 2012). DNase I is expensive to produce, and hence the use of DNase I on biofilms has been limited to medical applications, for example inner ear infections (Thornton et al., 2013) and wound biofilm control (Swartjes et al., 2013). More recently investigations have also been carried out into the activity of enzyme treatments with foodborne bacterial pathogens such as *Listeria monocytogenes*. For example, *L. monocytogenes* biofilms formed on stainless steel can be removed by both DNase I and Proteinase K treatments (Nguyen and Burrows, 2014), similar to reported here for *C. jejuni* biofilms.

The eDNA within the extracellular matrix appears to have multiple functions, depending on the bacterial species investigated. Previous research in *P. aeruginosa* biofilms has shown that eDNA can not only provide structural stability at early stages of biofilm formation (Whitchurch et al., 2002) but is also found to be localized to specific areas of the biofilm as it matures (Ma et al., 2009), again suggesting a structural role for eDNA in *P. aeruginosa* biofilm organization and expansion (Gloag et al., 2013), with DNase I treatment of developing biofilms leading to significant decreases in biofilm levels. DNA can be used as nutrient source by *E. coli*, *Shewanella*, and *P. aeruginosa* when exposed to phosphate and carbon deficient environments (Palchevskiy and Finkel, 2006; Pinchuk et al., 2008; Mulcahy et al., 2010). Since bacteria within the biofilm are typically immobilized, DNA could provide an easily obtainable food source. Finally, for naturally competent bacteria such as *C. jejuni*, the eDNA can contribute to the spread of genetic traits within populations, both in the biofilm and in the planktonic populations. Genetic material can be transferred within the biofilm either by direct cell to cell transmission or uptake of exogenous DNA. Conjugation within biofilms is a well reported phenomenon, with examples reported in mixed species oral biofilm models (Hannan et al., 2010), drinking water systems (Lisle and Rose, 1995) and within bacterial populations colonizing the nasopharynx (Marks et al., 2012). Recent work has shown that *C. jejuni* strains NCTC 11168 and 81–176 in microaerobic cultures are able to transfer genetic material between bacterial cells both within biofilms and planktonic suspension (Bae et al., 2014; Svensson et al., 2014). The work presented here shows that *C. jejuni* is also able to utilize exogenously added DNA for acquisition of genetic traits. This transfer is also able to occur in aerobic conditions, more closely resembling the conditions *C. jejuni* encounters while in the food chain.

We demonstrate here that eDNA is an important component of the *C. jejuni* extracellular matrix at all stages of maturation. This is in contrast to *P. aeruginosa*, which become less susceptible to DNase I treatment as the biofilm matures (Whitchurch et al., 2002). Some outer membrane and flagella proteins have been identified as been important in *C. jejuni* biofilm formation, but to date there has been little investigation of the extracellular matrix components themselves. *C. jejuni* produces a polysaccharide containing β1-3 and/or β1-4 linkages which is reactive to calcofluor white (McLennan et al., 2008), and hence further studies are required to distinguish between the roles of eDNA and other polysaccharides in *C. jejuni* biofilms.

Although eDNA has been shown to be present within the biofilms of many different bacteria, the mechanism of its release into the extracellular milieu is still under investigation. There are two main mechanisms of DNA release; secretion and cell lysis. Secretion of eDNA has been shown in several species, including *Neisseria gonorrhoeae* (Hamilton et al., 2005) and *P. aeruginosa* (Renelli et al., 2004). Although secretion of eDNA has been observed in some bacteria, it is widely accepted that lysis is a more common method of eDNA release (Wu and Xi, 2009). For instance, *Staphylococcus aureus* eDNA can be released via co-ordinated lysis of a subset of the population, controlled by quorum sensing (Mann et al., 2009). To date quorum sensing mechanisms have not been described in *C. jejuni* (He et al., 2008; Adler et al., 2014), and although it is possible that a yet unknown quorum sensing system controls co-ordinated eDNA release in *C. jejuni*, this will require further investigation. *P. aeruginosa* biofilms showed higher concentrations of eDNA within the biofilm when cultures were supplemented with salmon sperm DNA (Chiang et al., 2013), suggesting that some biofilm-forming bacteria are able to utilize eDNA from several sources. Our results suggest that although *C. jejuni* NCTC 11168 and 81116 are able to utilize exogenous DNA, this does not lead to a net increase in biofilm formation. In contrast, addition of eDNA to *C. jejuni* 81–176 biofilm cultures led to increased biofilm biomass (Svensson et al., 2014).

Another problem frequently encountered within food processing environments is the presence of food product debris. This presence of this debris on surfaces can lead to surface conditioning and increased bacterial attachment, as observed with chicken juice and *C. jejuni* (Brown et al., 2014). The attachment of *L. monocytogenes* to stainless steel surfaces is enhanced by surface pre-conditioning with fish and meat emulsions (Gram et al., 2007), and surface conditioning by chicken juice has been shown to enhance *C. jejuni* biofilm formation (Brown et al., 2014). Surface conditioning can also decrease the effectiveness of chemical cleaning products, leading to reduced killing or biofilm degradation (Gram et al., 2007). In heavily soiled environments broad spectrum enzymatic treatments may provide a useful and effective addition to current cleaning regimes, as they are able to degrade not only biofilm extracellular matrix, but potentially also the conditioning layer. Our results show that DNase I treatment is able to significantly reduce *C. jejuni* biofilms formed on surfaces conditioned with chicken juice, suggesting that DNase I treatment could provide a useful addition to current treatment regimens.

It should be noted that we found DNase I treatment had no effect on cell viability, only biofilm shedding. This is as expected since DNase I is only in contact with the DNA of the extracellular matrix, reducing the structural integrity of the colonies forming the biofilm, but is not able to cause a loss of viability in bacterial cells with intact membranes. This means that although the DNase I treatment provides a rapid and effective method of biofilm dispersal it would best be used in combination with antimicrobial treatments, ensuring effective biofilm degradation and bacterial inactivation.

In conclusion, eDNA is an essential component of the *C. jejuni* biofilm and its degradation results in a reduction of biofilm levels below detection levels (Tresse et al., 2006). Treatment of abiotic surfaces containing *C. jejuni* biofilms with DNase I also prevents re-establishment of biofilms, possibly allowing more efficient antimicrobial treatment. DNase I treatment is effective on food chain relevant surfaces and hence could provide a useful addition to current food chain cleaning regimes.

## Author contributions

HB, MR, RB, and AV designed the study. HB, KH, and MR performed the experimental work and analyzed the data. HB prepared the manuscript, and KH, MR, RB, and AV contributed to the final manuscript.

## Conflict of interest statement

Roy P. Betts is a full time employee of Campden BRI, which provided additional funding for the PhD-studentship of Helen L. Brown. The authors declare that the research was conducted in the absence of any commercial or financial relationships that could be construed as a potential conflict of interest.
