# Supplementary material for: Campylobacter jejuni biofilms contain extracellular DNA and are sensitive to DNase I treatment
Source: Front Microbiol. 2015 Jul 10;6:699. doi: 10.3389/fmicb.2015.00699 (PMC4498105; doi:10.3389/fmicb.2015.00699)
Supplement: Supplementary file 1 [file Presentation1.PDF]

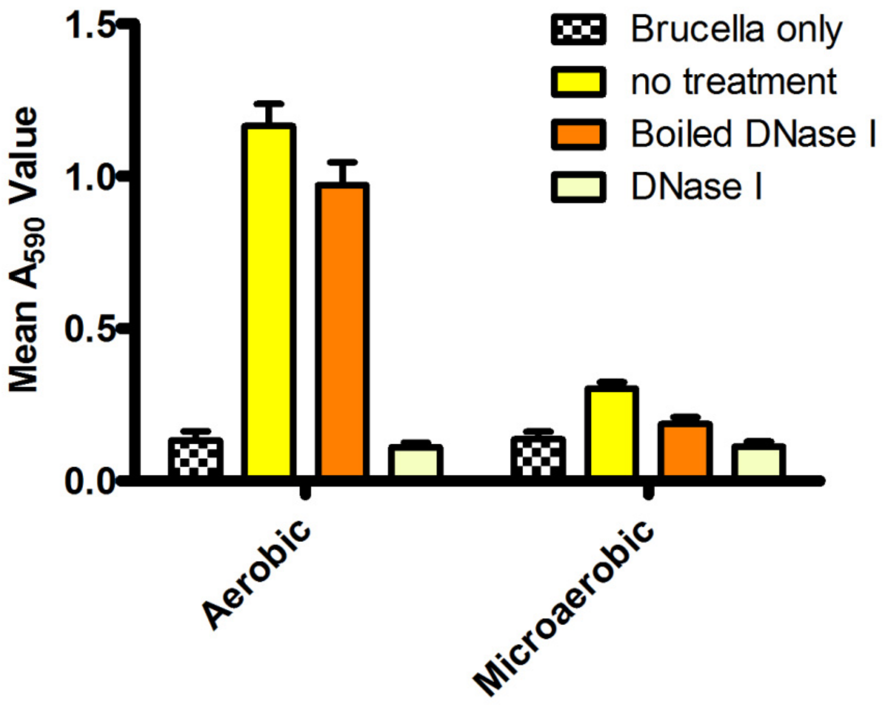

Fig. S1. Treatment of *C. jejuni* NCTC 11168 biofilms with boiled DNase does not statistically significantly reduce biofilm formation. Graph shows mean values of biofilm biomass, quantified by crystal violet staining. treatment of biofilms with heat inactivated DNase I (orange bars) gives comparable levels of staining to untreated NCTC 11168 biofilms (yellow bars). When DNase I is added to NCTC 11168 cultures at the start of the aerobic static incubation (white bar) there is a reduction in biofilm biomass which is comparable to that of a test tube containing only sterile Brucella medium (black and white bar). Error bars show SEM.
